# A prediction model for hospital mortality in patients with severe community-acquired pneumonia and chronic obstructive pulmonary disease

**DOI:** 10.1186/s12931-022-02181-9

**Published:** 2022-09-18

**Authors:** Dong Huang, Dingxiu He, Linjing Gong, Rong Yao, Wen Wang, Lei Yang, Zhongwei Zhang, Qiao He, Zhenru Wu, Yujun Shi, Zongan Liang

**Affiliations:** 1 Department of Respiratory and Critical Care Medicine, West China Hospital, Sichuan University, No. 37 Guoxue Alley, Chengdu, 610041 Sichuan China; 2 Institute of Clinical Pathology, Key Laboratory of Transplant Engineering and Immunology, NHC, West China Hospital, Sichuan University, No. 37 Guoxue Alley, Chengdu, 610041 Sichuan China; 3 Department of Emergency Medicine, The People’s Hospital of Deyang, Deyang, Sichuan China; 4 Department of Emergency Medicine, Emergency Medical Laboratory, West China Hospital, Sichuan University, Chengdu, Sichuan China; 5 Chinese Evidence-Based Medicine Center and CREAT Group, West China Hospital, Sichuan University, Chengdu, Sichuan China; 6 Department of Critical Care Medicine, West China Hospital, Sichuan University, Chengdu, Sichuan China

**Keywords:** Severe community-acquired pneumonia, Chronic obstructive pulmonary disease, Mortality, Risk factors, Nomogram

## Abstract

**Background:**

No personalized prediction model or standardized algorithm exists to identify those at high risk of death among severe community-acquired pneumonia (SCAP) patients with chronic obstructive pulmonary disease (COPD). The aim of this study was to investigate the risk factors and to develop a useful nomogram for prediction of mortality in those patients.

**Methods:**

We performed a retrospective, observational, cohort study in the intensive care unit (ICU) of West China Hospital, Sichuan University with all consecutive SCAP patients with COPD between December 2011 and December 2018. The clinical data within 24 h of admission to ICU were collected. The primary outcome was hospital mortality. We divided the patients into training and testing cohorts (70% versus 30%) randomly. In the training cohort, univariate and multivariate logistic regression analysis were used to identify independent risk factors applied to develop a nomogram. The prediction model was assessed in both training and testing cohorts.

**Results:**

Finally, 873 SCAP patients with COPD were included, among which the hospital mortality was 41.4%. In training cohort, the independent risk factors for hospital mortality were increased age, diabetes, chronic renal diseases, decreased systolic blood pressure (SBP), and elevated fibrinogen, interleukin 6 (IL-6) and blood urea nitrogen (BUN). The C index was 0.840 (95% CI 0.809–0.872) in training cohort and 0.830 (95% CI 0.781–0.878) in testing cohort. Furthermore, the time-dependent AUC, calibration plots, DCA and clinical impact curves indicated the model had good predictive performance. Significant association of risk stratification based on nomogram with mortality was also found (P for trend < 0.001). The restricted cubic splines suggested that estimated associations between these predictors and hospital mortality were all linear relationships.

**Conclusion:**

We developed a prediction model including seven risk factors for hospital mortality in patients with SCAP and COPD. It can be used for early risk stratification in clinical practice after more external validation.

**Supplementary Information:**

The online version contains supplementary material available at 10.1186/s12931-022-02181-9.

## Background

Community-acquired pneumonia (CAP), caused by a large variety of microorganisms including bacteria, respiratory viruses and fungi, is a common acute respiratory infection with high morbidity in all age groups worldwide [1]. Meanwhile, it is reported to be responsible for substantial mortality, with a third of patients dying within 1 year after being discharged from hospital [2]. One multicenter, population-based study released that 21% of pneumonia patients required intensive care, who were often considered to be severe CAP (SCAP) patients [3]. Recent advances in rapid diagnosis, microbiological investigation, appropriate and individualized antibiotic therapy, and management of complications have contributed to improving the outcomes of patients with SCAP. However, the mortality still remains high and is reported to be 25–50% globally [4].

Chronic obstructive pulmonary disease (COPD), the fourth leading cause of death worldwide, has also imposed such a heavy burden on healthcare systems. It affects close to 400 million people around the world [5]. Similarly, despite huge progress in the prevention and treatment, only few advances have been made to ameliorate the mortality or improve the prognosis of COPD patients. It is estimated that more than 3 million people die of COPD worldwide every year. Furthermore, it is predicted that COPD will remain a major health-care related problem for the next few decades [6].

SCAP is one of the most common infections in COPD patients. COPD represents a relevant risk factor for development of CAP, and one of the most frequently reported comorbid conditions in SCAP patients. Compared with SCAP patients without COPD, SCAP patients with COPD might have some distinct characteristics, including structural disruptions in the lung parenchyma, abnormal lung immunity and pulmonary function, worse respiratory failure, different lung microbiome and pathogen virulence, and increased risk of infection by Gram-negative bacilli or development of invasive pulmonary aspergillosis, etc. [7]. Moreover, one recent meta-analysis also found a positive association between COPD and increased 30-days mortality in patients with CAP (OR 1.84; 95% CI 1.06, 2.62) [8]. Hence, there is growing need for more researches and investigations in patients with SCAP and COPD.

Accurate and timely evaluation of risk of death on the basis of various predictors or risk factors is of great importance for early and effective therapy and management. To date, only a few relatively small studies have reported the risk factors for poor outcomes in SCAP patients with COPD. For instance, one study with 211 patients with COPD and CAP requiring intensive care unit (ICU) admission reported that bilateral infiltration (OR 13.92; 95% CI 2.94–65.84) and longer duration of invasive mechanical ventilation (OR 1.11; 95% CI 1.01–1.22) were associated with increased in-hospital mortality [9]. However, no personalized prediction model or standardized algorithm exists to identify those at high risk of death among SCAP patients with COPD. The increasing rates of COPD in SCAP patients highlights the importance of a thorough assessment with an accurate and useful tool when managing those patients. The aim of this study was to investigate the risk factors and to develop a useful nomogram for prediction of hospital mortality in those patients.

## Methods

### Study design and cohort

We performed a retrospective, observational, cohort study in the ICU with over 200 beds in a large tertiary-care teaching hospital in Chengdu city, Sichuan province, China in accordance with the amended Declaration of Helsinki. The study was approved by the West China Hospital of Sichuan University Biomedical Research Ethics Committee (No. 2021-828). The requirement to obtain informed consent in this analysis was waived due to the retrospective noninterventional design. All analyses were conducted in accordance with the Transparent Reporting of a Multivariable Prediction Model for Individual Prognosis or Diagnosis statement [10].

For construction and validation of the nomogram, we divided the patients into training and testing cohorts (70% versus 30%) randomly to ensure comparability between the cohorts. With approximately 20 putative variables potentially related to mortality, the minimum sample size required 200 deaths to follow the principle of at least 10 outcome events per variable (EPV) in the regression analysis [11]. Considering that the mortality of SCAP was approximately 40% in previous reports [4], the sample size of training cohort was estimated to be approximately 500. And therefore, the overall sample size was at least 715. Hence, all consecutive SCAP patients with COPD admitted to ICU between December 2011 and December 2018 were included in the current study.

According to the Infectious Diseases Society of America (IDSA)/American Thoracic Society (ATS) guidelines, SCAP was defined as fulfilment of at least 1 major criterion (septic shock with need for vasopressors; respiratory failure requiring mechanical ventilation) or 3 minor criteria (respiratory rate ≥ 30 breaths/min; PaO_2_/FiO_2_ ratio ≤ 250; multilobar infiltrates; confusion/disorientation; blood urea nitrogen level ≥ 20 mg/dL; white blood cell count < 4000 cells/µL; platelet count < 100,000/µL; core temperature < 36 °C; hypotension requiring aggressive fluid resuscitation) [12]. COPD was diagnosed based on medical history, clinical manifestation and the presence of persistent airflow limitation with a post-bronchodilator FEV1/FVC less than 0.70 on spirometry according to the Global Initiative for Chronic Obstructive Lung Disease (GOLD) report [13].

The exclusion criteria were as follows: (1) residents of long-term care facilities and/or nursing homes; (2) prior hospitalization within 30 days of study enrollment; (3) unclear outcomes; (4) severe immunosuppression defined according to a consensus statement [14], including active solid or hematological malignancy; HIV infection with a CD4 T-lymphocyte count < 200 cells/mL; receiving corticosteroid therapy with a dose ≥ 20 mg prednisone or equivalent daily for ≥ 14 days or a cumulative dose > 600 mg of prednisone; receiving cancer chemotherapy, biological immune modulators, disease-modifying antirheumatic drugs or other immunosuppressive drugs; (5) only the first admission was included if the patient had repeated admission.

All patients received standard care and antibiotic therapy to the discretion of the ICU attending physician and based on the CAP guidelines [12].

### Study outcomes and measurements

The following clinical data within 24 h of admission to the ICU were collected anonymously from electronic medical records: demographic characteristics, comorbidities, vital signs, and laboratory examinations including hematological data, biochemical parameters, inflammatory markers, coagulation indicators, etc. All patients’ data were anonymized and de-identified. The first value was recorded for analysis if any laboratory examination was repeated more than once within 24 h of admission.

Two experienced physicians reviewed the medical records and completed the data collection by using a standardized data collection form independently. Data were checked by a third reviewer if there was any disagreement.

Patient follow-up was until hospital discharge. The primary outcome established for this study was hospital mortality.

### Statistical analysis

Data were analyzed using IBM SPSS Statistical version 23.0 (SPSS, Chicago, IL, USA) and R software 4.1.2 (R Foundation for Statistical Computing). For standard analyses, a two-sided p < 0.05 was considered to indicate statistical significance. Data are presented as median (interquartile range, IQR) for continuous variables and number (percentage) for categorical variables as appropriate. The nonparametric Kruskal–Wallis test, chi-square analysis and Fisher’s exact test were used to test for differences between groups as appropriate. All baseline clinical data were compared between training cohort and testing cohort. Multiple imputation (MI) was used to account for missing data by using Bayesian methods in SPSS.

In the training cohort, the potential variables with P < 0.05 in univariate logistic regression analysis were included in the multivariate analysis with stepwise forward selection to identify independent risk factors for hospital mortality. The results were reported as odds ratios (ORs) and 95% confidence intervals (95% CIs). A simple nomogram based on independent risk factors was developed to predict individual probability of death. The prediction model was assessed using the concordance index (C index), area under receiver-operating characteristic (ROC) curve (AUC), area under time-dependent ROC curve (time-dependent AUC), calibration curves, decision curve analysis (DCA) and clinical impact curves in both training and testing cohorts [15–17].

Then, all patients were divided into three groups with different risks of mortality (low, moderate and high risk) according to nomogram to increase its clinical utility. The P values and the P for trend through the groups were calculated to further evaluate the nomogram. A Spearman correlation analysis was carried out to test the correlations of the continuous variables among the predictors. Finally, we also applied restricted cubic splines to estimate the possible non-linear associations between risk factors as continuous variables and mortality [18]. It was performed using the Regression Modeling Strategies (rms) package in R. The locations of the knots were set at the 10th, 50th and 90th percentiles with three knots. Analyses were multivariate-adjusted for all independent risk factors.

## Results

### Characteristics of SCAP patients with COPD

**Fig. 1:**
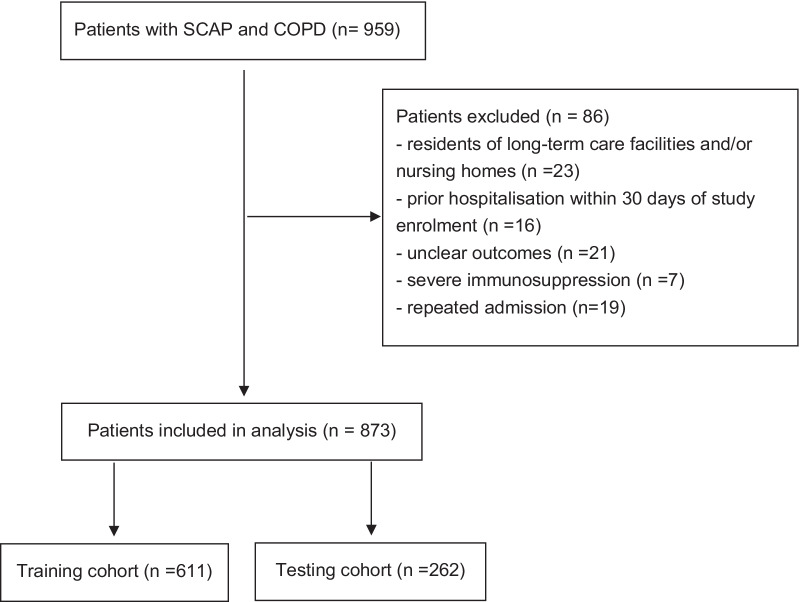
Study population. *SCAP* severe community-acquired pneumonia; *COPD* chronic obstructive pulmonary disease

**Fig. 2:**
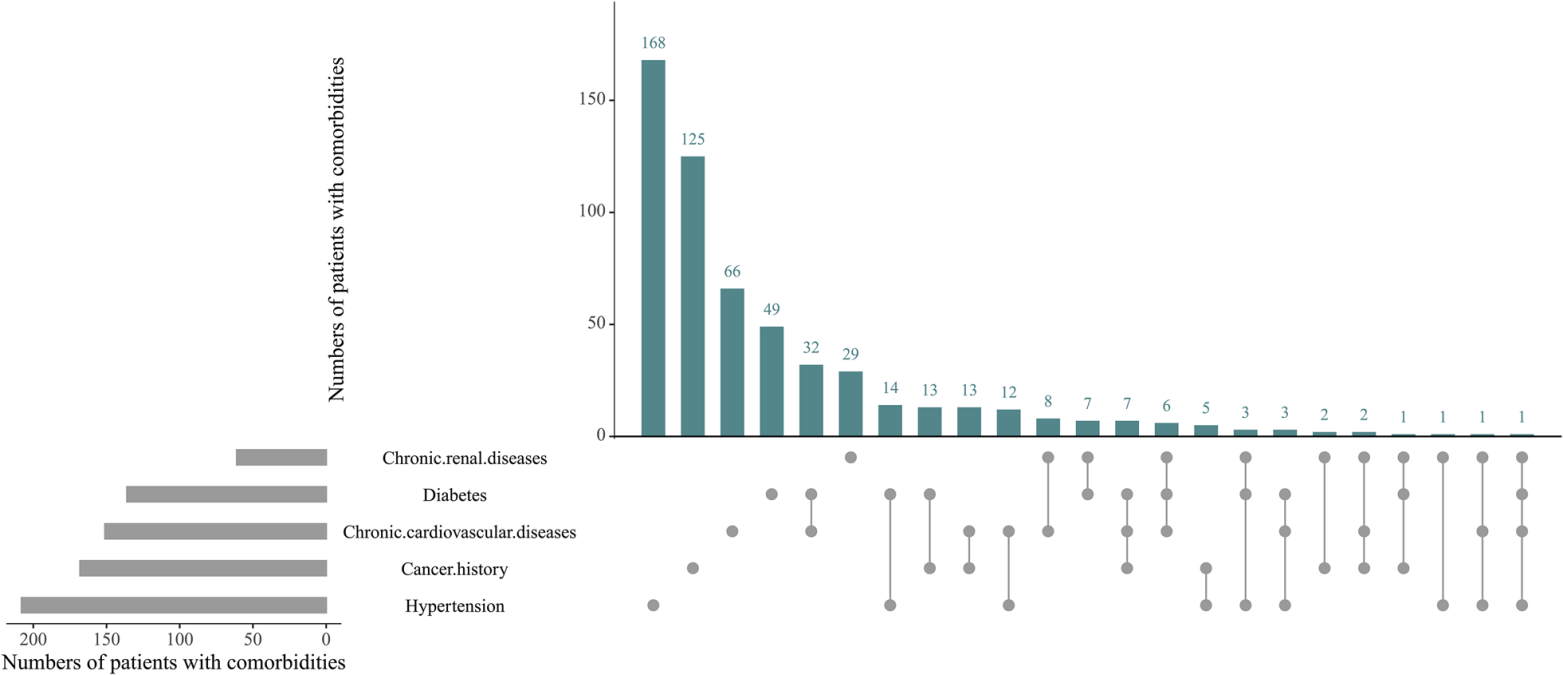
The common coexisting medical conditions in SCAP patients with COPD. *SCAP* severe community-acquired pneumonia; *COPD* chronic obstructive pulmonary disease

In total, 959 patients with SCAP and COPD between December 2011 and December 2018 were retrospectively screened. Then, 86 patients were excluded according to the exclusion criteria (Fig. 1). Among the remaining 873 SCAP patients with COPD, the median age was 77 years old (IQR 69,83) and 619 (70.9%) of patients were male. The patients’ comorbidities were summarized in Fig. 2. Hypertension, cancer history, chronic cardiovascular diseases, diabetes, chronic cardiovascular diseases and diabetes, and chronic renal diseases were the six most common coexisting medical conditions. The ICU mortality and hospital mortality was 36.5% (319 patients) and 41.4% (361 patients), respectively.

Finally, 611 patients were randomized to training cohort and 262 were assigned into testing cohort. Detailed comparison of clinical data between training cohort and testing cohort was shown in Additional file 1: Table S1 and Table S2. There were no significant differences in the features of demographic characteristics, comorbidities, vital signs, laboratory examinations and clinical outcomes.

### Construction of nomogram

In training cohort, 17 variables were identified in the univariate logistics regression model. After that, multivariate analysis revealed that the independent risk factors for hospital mortality were increased age, diabetes, chronic renal diseases, decreased systolic blood pressure (SBP), and elevated fibrinogen, interleukin 6 (IL-6) and blood urea nitrogen (BUN). The detailed ORs and 95%CIs in univariate and multivariate analysis were summarized in Table 1.

**Table 1:** Risk factors associated with hospital mortality in training cohort

Risk factors	Univariate analysis		Multivariate analysis	
	OR (95% CI)	P	OR (95% CI)	P
Demographic characteristics				
Age	1.022 (1.005, 1.039)	0.012	1.025 (1.003, 1.047)	0.025
Comorbidities				
Diabetes	1.618 (1.052, 2.489)	0.028	1.974 (1.156, 3.371)	0.013
Chronic renal diseases	2.378 (1.242, 4.554)	0.009	2.708 (1.182, 6.204)	0.019
Chronic cardiovascular diseases	1.568 (1.047, 2.347)	0.029		
Chronic cerebrovascular diseases	5.454 (1.506, 19.755)	0.010		
Vital signs				
Systolic blood pressure (mmHg)	0.979 (0.973, 0.985)	< 0.001	0.980 (0.972, 0.988)	< 0.001
Diastolic blood pressure (mmHg)	0.990 (0.980, 1.000)	0.047		
Laboratory examinations				
Fibrinogen (g/L)	1.375 (1.250, 1.512)	< 0.001	1.305 (1.153, 1.476)	< 0.001
IL-6 (pg/mL)	1.013 (1.010, 1.015)	< 0.001	1.012 (1.009, 1.014)	< 0.001
BUN (mmol/L)	1.081 (1.054, 1.109)	< 0.001	1.058 (1.024, 1.094)	0.001
Neutrophil (×10^9^/L)	1.025 (1.001, 1.050)	0.043		
Creatinine (µmol/L)	1.002 (1.000, 1.004)	0.036		
Myoglobin (ng/mL)	1.000 (1.000, 1.001)	0.041		
Troponin T (ng/L)	1.002 (1.000, 1.004)	0.017		
Glucose (mmol/L)	1.069 (1.023, 1.116)	0.003		
Platelet (×10^9^/L)	0.998 (0.997, 1.000)	0.030		
Lactate (mmol/L)	1.113 (1.017, 1.217)	0.019		

Data were calculated using logistics regression model

*OR* odds ratio; *95% CI* 95% confidence interval; *IL-6* interleukin-6; *BUN* blood urea nitrogen

**Fig. 3:**
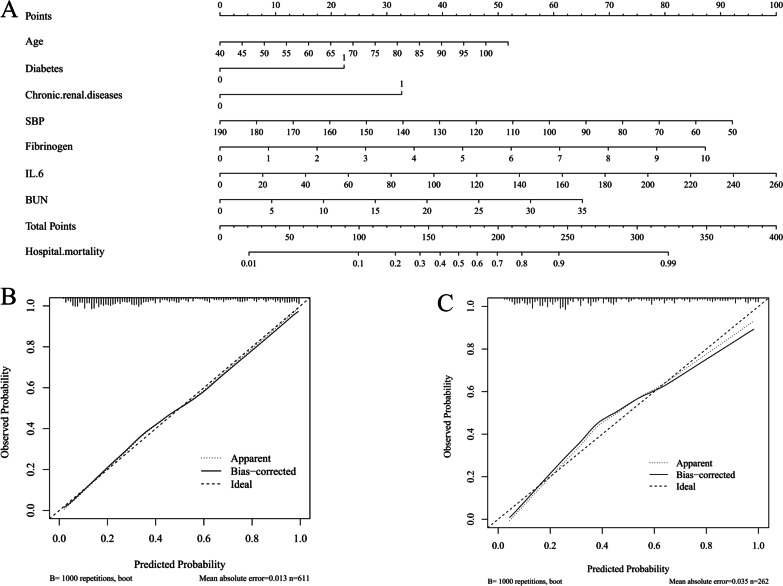
** A** The nomogram for hospital mortality in SCAP patients with COPD. Age (years old); Diabetes and Chronic renal diseases (1: yes; 0: no); SBP (Systolic blood pressure, mmHg); Fibrinogen (g/L); IL-6 (interleukin-6, pg/mL) BUN (blood urea nitrogen, mmol/L). **B** Calibration curve of nomogram in training set. **C** Calibration curve in testing set. *SCAP* severe community-acquired pneumonia; *COPD* chronic obstructive pulmonary disease

Therefore, the above seven factors were used to construct the prediction model. To calculate individual patient scores and corresponding risk of death, a simple nomogram is available (Fig. 3A). According to the nomogram, each predictor corresponded to a point at each value. The total point was the sum of the points of seven predictors for each patient. The relationship between the total point and the probability of death was shown on the bottom of the nomogram.

### Assessment of nomogram

**Fig. 4:**
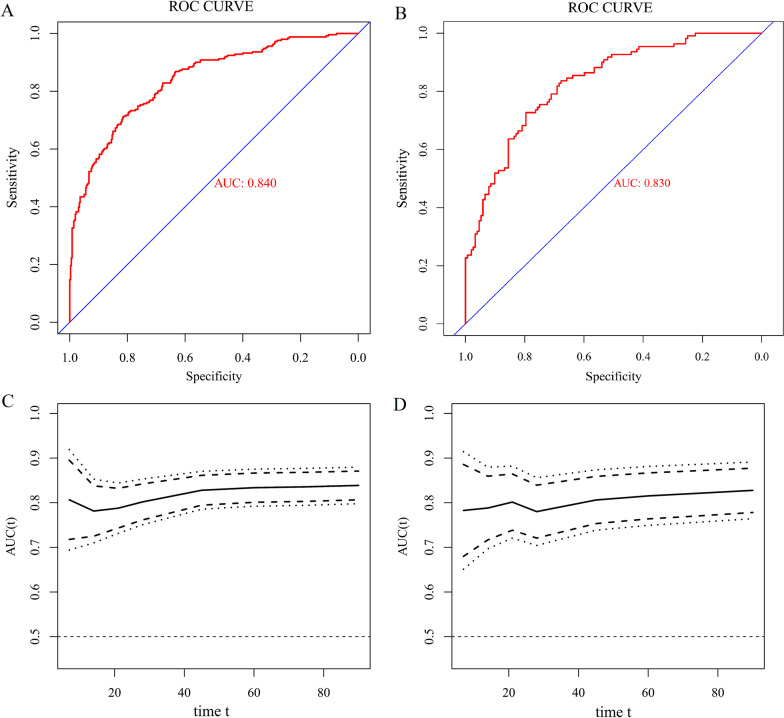
** A** The ROC curve of nomogram for training cohort. **B** The ROC curve for testing cohort. **C** The time-dependent AUC in training cohort (days). **D** The time-dependent AUC in testing cohort (days). *ROC* receiver-operating characteristic; *AUC* area under ROC curve

The C index was 0.840 (95% CI 0.809–0.872) in training cohort and 0.830 (95% CI 0.781–0.878) in testing cohort, which indicated that the prediction model had good predictive discrimination. The ROC curves and AUCs for training cohort and testing cohort were displayed in Fig. 4A and B. Furthermore, the time-dependent AUC of model in training cohort and testing cohort were shown in Fig. 4C and D, respectively. The time-dependent AUC was around 0.80 for the prediction of death within 90 days after admission in both the training cohort and testing cohort, indicating favorable and robust discrimination of the model.

In Fig. 3B and C, the calibration plots suggested a high consistency, which demonstrated that the model’s predicted probabilities were close to the observed actual probabilities. The bias corrected C index was 0.834 and 0.813 in training cohort and testing cohort, respectively.

**Fig. 5:**
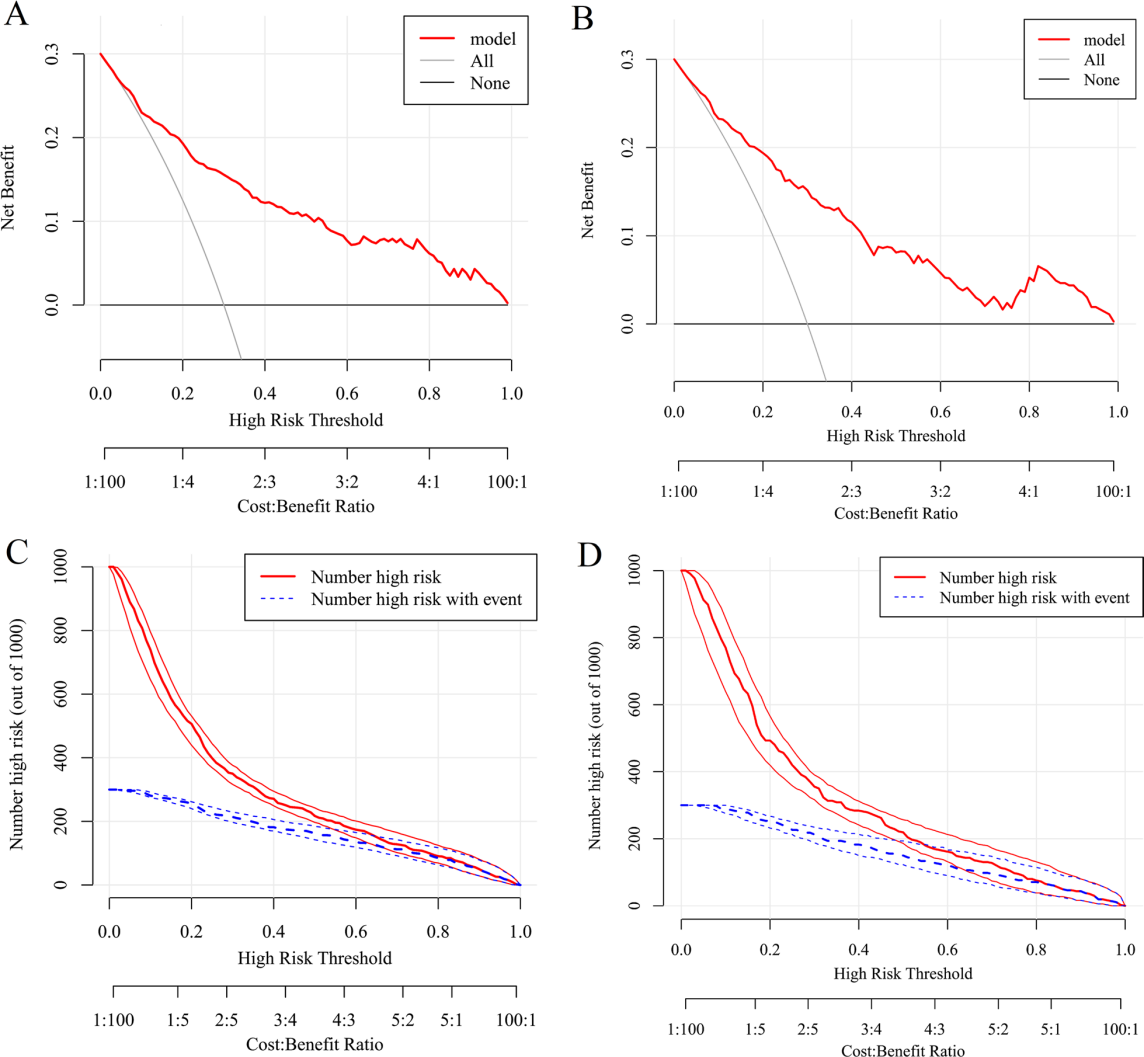
** A** The DCA of nomogram for training cohort. **B** The DCA for testing cohort. **C** The clinical impact curve for training cohort. **D** The clinical impact curve for testing cohort. *DCA* decision curve analysis

The DCA compared the net benefit of each score across different thresholds and showed that the majority of the threshold probabilities had great net benefit (Fig. 5A, B). In addition, we further plotted clinical impact curves to predict improved probability stratification for a population size of 1000. It showed that the predicted probability coincided well with the actual probability in both training cohort and testing cohort (Fig. 5C, D).

### Clinical utility of nomogram and predictors

To further investigate the clinical utility of prediction model, all patients were divided into three groups according the total points calculated from nomogram: low risk (total points: under 150), moderate risk (from 150 to 200) and high risk (above 200). Compared with patients in low-risk group, the ORs (95% CIs) for hospital mortality of patients in moderate-risk and high-risk group were 4.102 (2.893, 5.815) and 22.130 (13.266, 36.919), respectively (P for trend < 0.001). (Table 2).

**Table 2:** The risk stratification of SCAP-COPD patients in terms of nomogram

Risk group	Number of patients (%)	Total points	OR (95% CI)	P value	P for trend
Low risk	528 (60.5)	< 150	1 (reference)	–	
Moderate risk	192 (22)	150–200	4.102 (2.893, 5.815)	< 0.001	
High risk	153 (17.5)	> 200	22.130 (13.266, 36.919)	< 0.001	< 0.001

Data were calculated using logistics regression model

*OR* odds ratio; *95% CI* 95% confidence interval

**Fig. 6:**
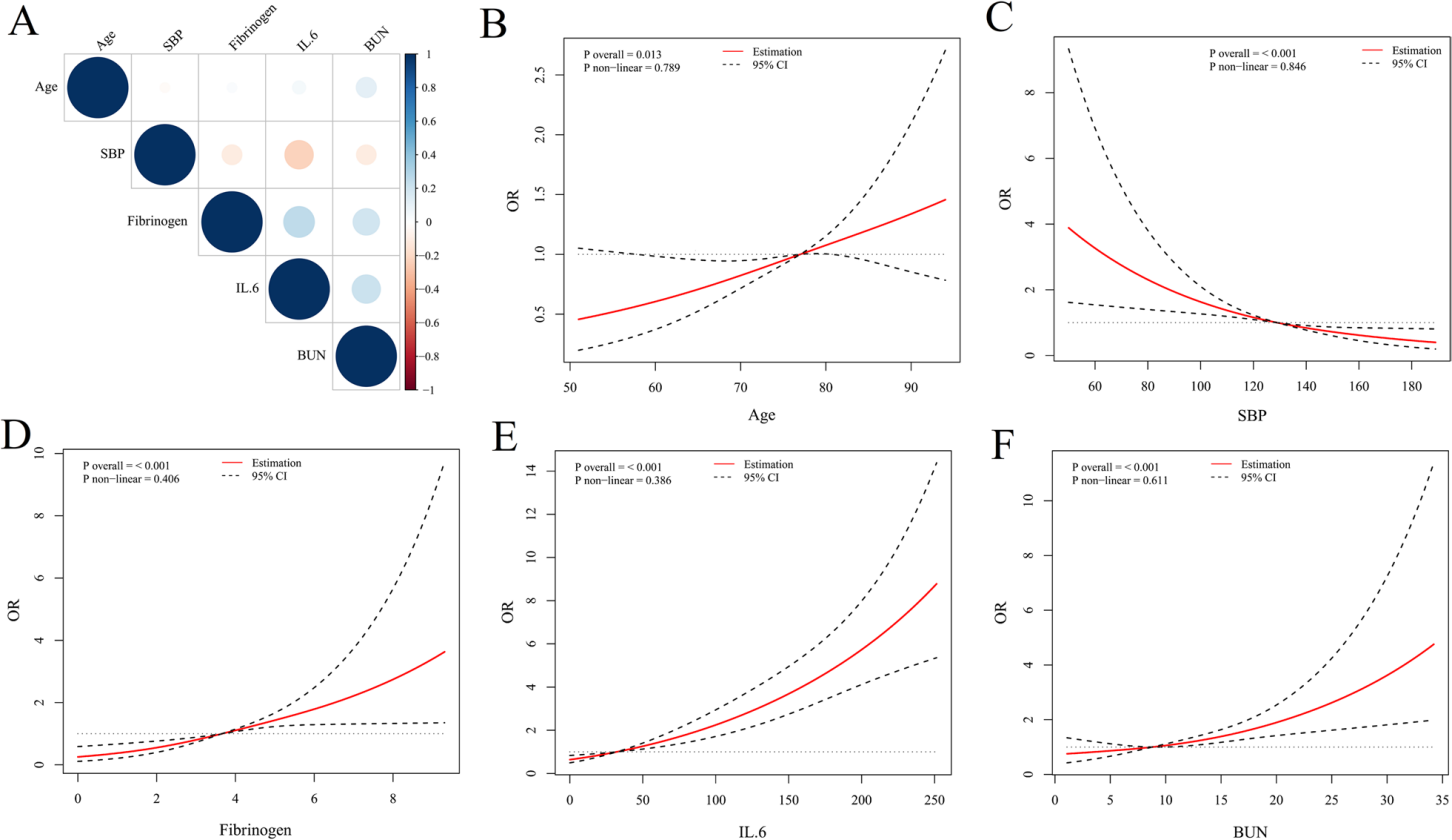
** A** Spearman correlation analysis. **B–F** The restricted cubic splines with three knots. The horizontal dashed line represents the reference OR of 1.0. The model was multivariate-adjusted for age, diabetes, chronic renal diseases, systolic blood pressure (SBP), fibrinogen, interleukin 6 (IL-6) and blood urea nitrogen (BUN). *OR* odds ratio; *95% CI* 95% confidence interval

In Fig. 6A, the fibrinogen, IL-6 and BUN were positively correlated with each other. Meanwhile, the systolic blood pressure was negatively correlated with them (P for Spearman correlation analysis < 0.05). However, the correlations of age with them were not significant.

As shown in Fig. 6B–F, the estimated associations between these predictors and hospital mortality were all linear relationships (P for non-linear > 0.05), which further demonstrated the predictive performances and prognostic accuracies of these predictors as continuous variables.

## Discussion

To the best of our knowledge, this is the first practical prediction model to identify patients at risk of death in those with SCAP and COPD specifically. Our model integrates various basic clinical characteristics, including age, comorbidities, vital signs and laboratory examinations, indicating that the comprehensive evaluation based on these predictors is essential. The C indices and time-dependent AUCs of the nomogram when applied to the training and testing cohorts were similar and both approximately 0.8, which demonstrated the performance was relatively ideal. The score can be calculated by hand according to nomogram with routine parameters tested in the laboratory. Hence, it is rapid, cost-effective and can be easily implemented in clinical practice.

The independent risk factors of death in CAP patients with COPD varied widely in the existing literature. For example, Bonnesen et al. included 243 CAP patients with COPD and found that the factors related to mortality were age, premorbid condition, CURB-65 score, pleural effusion and multi-lobular infiltrate [19]. In another research, aspiration (OR 5.203; 95% CI 1.443, 18.757), D-dimer > 2.0 µg/mL (OR 5.026; 95% CI 1.395, 18.108) and CURB-65 ≥ 3 (OR 23.299; 95% CI 6.246,  86.903) were risk factors of in-hospital mortality in 230 CAP patients comorbid with COPD [20]. Multilobar pneumonia (OR 2.883; 95% CI 1.299–6.399), *Pseudomonas aeruginosa* pneumonia (OR 19.091; 95% CI 4.326–84.256) and high-risk PSI classes (OR 10.316; 95% CI 1.691–62.946) were also found to be independent risk factors for case-fatality rate in a prospective cohort of CAP patients with COPD [21]. Moreover, Shin et al. found the serum hemoglobin concentration (HR 0.759; 95% CI 0.616, 0.936) and albumin level (HR 0.429; 95% CI 0.185, 0.995) were significantly associated with 180-day mortality in 134 acute exacerbation of COPD (AECOPD) patients with CAP [22]. The inconsistency regarding diverging results across prior studies could be attributable to a combination of factors such as study design, population, severity of CAP and treatments. The study from Cilli et al. only included CAP patients in the ICU [9]. However, researchers did not assess the prediction performances of risk factors. Besides, few prior studies focused on the weight of each risk factors for outcomes. Therefore, it is likely that this study has several advantages or more important clinical implications compared with previous studies. First, we had a larger population with only SCAP patients in ICU enrolled, which is representative of the real-world pneumonia patient cohort that has the highest mortality. Then, it has been suggested that the biomarkers are a cornerstone in the management of SCAP to decrease treatment failure [23]. Considering that the combination of biomarkers would be of greater use than individual predictor, we developed a prediction model. Afterwards, we also evaluated and validated the model with several statistical methods. Third, there is no consensus on the optimal cut-off values of these predictors in SCAP patients. Therefore, they were included in the model as continuous variables. Moreover, we carefully investigated the prognostic accuracies and clinical utilities of them via correlation analysis and restricted cubic splines.

Patients with advanced age, chronic renal diseases, decreased systolic blood pressure, elevated BUN are also classified as high-risk population when conventional score calculations are applied in SCAP patients, such as CURB-65, pneumonia severity index (PSI), Sequential Organ Failure Assessment (SOFA) score and Acute Physiology and Chronic Health Evaluation (APACHE) II tool. As observed clinically and previously reported, our nomograms show that diabetes, a common comorbidity of COPD, is associated with worse prognosis. Several factors might be responsible for the mechanisms. Previous evidence suggested that both COPD and impaired lung function, especially restricted ventilation dysfunction, could increase the risk of diabetes as a consequence of systemic inflammatory processes [24]. In addition, treatment with corticosteroids in COPD could possibly lead to a variety of side effects, such as worsening hyperglycemia and deterioration of diabetes control [25]. And reversely, diabetes can worsen the prognosis of COPD due to the direct effects of hyperglycemia on lung physiology, inflammation and susceptibility to bacterial infection [26]. Moreover, diabetes is potentially associated with a wide spectrum of complications which negatively affect the prognosis of COPD, such as pulmonary hypertension [27]. Therefore, careful evaluation and management should be conducted in SCAP patients with COPD and diabetes due to the possible poor prognosis. IL-6 is involved in various hematopoietic, immune, and inflammatory responses. Therefore, it has been widely used as an early sensitive prognostic biomarker and a predictor of treatment failure and mortality in CAP [28]. He et al. found that IL-6 (hazard ratio [HR] 1.001; p = 0.001) could serve as independent predictors of 30-day mortality for CAP after adjusting for clinical data, including age, bilateral lung infection, procalcitonin, CURB-65, PSI, etc. [29]. Similarly, as an inflammatory marker and coagulation factor which is synthesized by hepatocytes and circulating in the bloodstream, the concentrations of fibrinogen are rapidly elevated in tissue injury, infection, inflammation, etc. It could also be used in the CAP severity evaluation [30]. Their prognostic values have also been investigated in COPD. In a meta-analysis with 61 studies in COPD, increased levels of IL-6 were associated with hospitalization (standardized mean difference [SMD] 0.12, 95%CI 0.04–0.20) and higher levels of fibrinogen were also associated with exacerbation (SMD 0.23 g/dL, 95%CI 0.14–0.33) and mortality (HR 3.13 per twofold increase, 95%CI 2.14–4.57) [31]. Zhou et al. conducted another meta-analysis with 45 studies and found a graded, concentration-dependent, significant relation between higher circulating fibrinogen and more severity of COPD [32]. Hence, it is plausible that elevated admission IL-6 and fibrinogen both are associated with hospital mortality in SCAP patients with COPD.

Some factors, such as increased creatinine and Troponin T, were associated with the mortality in univariate analysis. Nonetheless, the associations disappeared when adjusting for other risk factors. However, we should be cautious when explaining this conclusion because results from the existing literature on patients with SCAP or COPD are inconsistent with regard to whether they are associated with survival [33–36]. Future studies should address whether they could improve the evaluation and prediction of outcomes in SCAP patients with COPD.

The existing reports believed that risk stratification and early identification might contribute to optimizing the management of SCAP, with potential reduction of mortality [37]. Early assessment via prediction model could be instrumental to quantify in advance an individual patient’s risk of death when planning the therapies. On the other hand, the identification of patients at highest risk is pivotal to implement early measures and improve prognosis. The nomograms could be utilized as a complementary tool for decision making in clinical practice, or for SCAP-COPD patient selection in future studies on the basis of their risk stratification using the risk grouping. However, we acknowledge that some issues remain to be addressed. First, in our study, the diagnosis of COPD may lack strictness. It was difficult to determine the severity of COPD patients or to stratify them according to exacerbation histories, lung functions and symptoms from the data available. Thus, the identified independent risk factors need to be confirmed in COPD patients with different clinical characteristics. In addition, the nomogram might also have decreased predicting value in some specific subgroups of COPD patients. Then, the patients in present study are a little older (median age: 77 years old) compared to those SCAP patients in previous observational studies [19–22]. One leading cause is that we only strictly included confirmed SCAP-COPD patients because COPD is considered as an age-related disease. However, it is worth noting that different baseline characteristics existing among studies could result in diverse conclusions. Thus, large-scale, multicenter, prospective studies are desirable to validate, recalibrate, improve discriminative capacity and increase the generalizability of our prediction model. Besides, further information is needed to shed light on deeper understanding of pathophysiological mechanisms of SCAP patients with COPD. For instance, more efforts could be dedicated to investigate the impacts of various coexisting medical conditions, such as chronic cardiovascular diseases and diabetes, on the mortality of SCAP patients with COPD. Future researches should also consider the prognostic effects of more pre-admission individual features, including smoking status, vaccination history, prior antibiotic treatment and corticosteroid use, etc. Meanwhile, it is still controversial whether identified pathogens or imaging findings are related to the severity or mortality in those patients.

The main limitation of the current study is the single-center, retrospective design with selection bias. Then, the missing data might have reduced the effective sample size, caused inevitable bias and threatened the validity of the study. Third, although a number of potential risk factors have been analyzed, we cannot exclude that some unadjusted confounders could have affected the results or some untested variables would further improve the model. The model can be updated when more multicentric data become available.

## Conclusion

In conclusion, we developed a prediction model for hospital mortality in patients with SCAP and COPD. The nomogram including seven risk factors with favorable predictive accuracy, discrimination, and clinical utility allows simple and rapid individual patient risk estimates. It can be used at admission of ICU to predict mortality to prompt early risk stratification and actionable measures in clinical practice after more external validation.

## Supplementary Information

**Additional file 1: Table S1.** Baseline characteristics of SCAP-COPD individuals in training cohort and testing cohort. **Table S2.** Numbers and percentages of missing values for each variable.

## Data Availability

The datasets used and/or analysed during the current study are available from the corresponding author on reasonable request.

## Abbreviations

SCAP: Severe community-acquired pneumonia
COPD: Chronic obstructive pulmonary disease
ICU: Intensiv care unit
OR: Odds ratio
95% CI: 95% confidence interval
SBP: Systolic blood pressure
IL-6: Interleukin-6
BUN: Blood urea nitrogen
