# Supplementary material for: A prediction model for hospital mortality in patients with severe community-acquired pneumonia and chronic obstructive pulmonary disease
Source: Respir Res. 2022 Sep 18;23:250. doi: 10.1186/s12931-022-02181-9 (PMC9482754; doi:10.1186/s12931-022-02181-9)
Supplement: Supplementary file 1 — Additional file 1: Table S1. Baseline characteristics of SCAP-COPD individuals in training cohort and testing cohort. Table S2. Numbers and percentages of missing values for each variable. [file 12931_2022_2181_MOESM1_ESM.docx]

Table S1. Baseline characteristics of SCAP-COPD individuals in training cohort and testing cohort.

| Variables | Overall (n=873) | training cohort (n=611) | testing cohort (n=262) | p |
| --- | --- | --- | --- | --- |
| Demographic characteristics |  |  |  |  |
| Age (years old) | 77 (69,83) | 77 (69,83) | 77 (69,83) | 0.988 |
| Sex: male (%) | 619 (70.9) | 432 (70.7) | 187 (71.4) | 0.906 |
| Comorbidities |  |  |  |  |
| Cancer history (%) | 168 (19.2) | 127 (20.8) | 41 (15.6) | 0.095 |
| Chronic hematological diseases (%) | 29 (3.3) | 19 (3.1) | 10 (3.8) | 0.743 |
| Diabetes (%) | 136 (15.6) | 100 (16.4) | 36 (13.7) | 0.38 |
| Chronic hepatic diseases (%) | 27 (3.1) | 23 (3.8) | 4 (1.5) | 0.124 |
| Chronic renal diseases (%) | 61 (7.0) | 41 (6.7) | 20 (7.6) | 0.73 |
| Chronic cardiovascular diseases (%) | 151 (17.3) | 118 (19.3) | 33 (12.6) | 0.021 |
| Hypertension (%) | 208 (23.8) | 142 (23.2) | 66 (25.2) | 0.594 |
| chronic cerebrovascular diseases (%) | 16 (1.8) | 14 (2.3) | 2 (0.8) | 0.205 |
| Vital signs |  |  |  |  |
| Respiratory rate (breath/min) | 20 (16, 24) | 20 (16, 24) | 19 (16, 23) | 0.263 |
| Systolic blood pressure (mmHg) | 129 (109,148) | 129 (110,147) | 128 (108,149) | 0.871 |
| Diastolic blood pressure (mmHg) | 68.00 (58.75, 80.00) | 67.00 (58.75, 79.00) | 69.00 (58.75, 82.00) | 0.194 |
| Temperature (°C) | 36.6 (36.3, 37.0) | 36.6 (36.3, 37.0) | 36.5 (36.2, 37.0) | 0.1 |
| Heart rate (beat/min) | 96.00 (80.00, 110.00) | 95.00 (80.00, 109.00) | 98.00 (82.75, 112.25) | 0.174 |
| Unconsciousness or insanity | 146 (16.7) | 96 (15.7) | 50 (19.1) | 0.261 |
| Laboratory examinations |  |  |  |  |
| White blood cell (×10 ^9^ /L) | 10.11 (6.98, 13.88) | 10.00 (6.87, 13.82) | 10.57 (7.34, 14.35) | 0.177 |
| Neutrophil (×10 ^9^ /L) | 8.22 (5.28, 12.20) | 8.20 (5.36, 12.31) | 8.34 (5.08, 11.90) | 0.999 |
| Lymphocyte (×10 ^9^ /L) | 0.77 (0.47, 1.21) | 0.77 (0.47, 1.21) | 0.78 (0.46, 1.17) | 0.849 |
| Albumin (g/L) | 31.40 (28.40, 34.80) | 31.20 (28.20, 34.70) | 31.75 (28.52, 35.38) | 0.233 |
| BUN (mmol/L) | 8.8 (6,13.67) | 8.8 (5.93, 13.18) | 8.86 (6.1,15.37) | 0.185 |
| D-dimer (mg/L) | 3.30 (1.83, 7.48) | 3.31 (1.83, 7.83) | 3.19 (1.93, 6.33) | 0.815 |
| Total bilirubin (μmol/L) | 10.00 (6.80, 14.90) | 10.00 (6.90, 14.93) | 10.00 (6.60, 14.53) | 0.589 |
| Direct bilirubin (μmol/L) | 5.10 (3.20, 7.90) | 5.10 (3.27, 7.90) | 5.10 (3.20, 7.70) | 0.945 |
| Globulin (g/L) | 24.40 (21.15, 28.70) | 24.30 (21.20, 28.20) | 25.00 (20.72, 29.20) | 0.514 |
| Monocyte (×10 ^9^ /L) | 0.42 (0.25, 0.65) | 0.41 (0.25, 0.64) | 0.42 (0.27, 0.65) | 0.806 |
| Hemoglobin (g/L) | 108.00 (91.50, 125.00) | 108.00 (93.00, 127.00) | 107.00 (87.25, 125.00) | 0.174 |
| APTT (s) | 33.30 (28.75, 40.00) | 33.45 (29.08, 40.30) | 32.80 (28.00, 39.60) | 0.186 |
| PT (s) | 13.1 (12.0, 14.4) | 13.0 (12.0, 14.3) | 13.1 (12.0, 14.7) | 0.492 |
| Fibrinogen (g/L) | 3.66 (2.60,4.80) | 3.69 (2.61,4.82) | 3.51 (2.59,4.69) | 0.345 |
| Creatinine (μmol/L) | 71.00 (52.00, 106.45) | 70.00 (51.40, 105.00) | 73.00 (53.00, 111.00) | 0.241 |
| Myoglobin (ng/mL) | 97.01 (46.77, 232.80) | 96.47 (46.35, 224.60) | 97.59 (47.60, 271.68) | 0.865 |
| ALT (IU/L) | 19.00 (12.00, 38.00) | 19.00 (12.00, 38.00) | 19.50 (12.00, 37.75) | 0.63 |
| AST (IU/L) | 27 (19, 45) | 27 (20, 45) | 27 (18, 46) | 0.588 |
| Troponin T (ng/L) | 39.00 (22.23, 79.53) | 38.30 (22.40, 77.65) | 41.00 (21.28, 82.42) | 0.625 |
| BNP (pg/mL) | 1533.00 (601.50, 4403.00) | 1443.00 (597.00, 4238.00) | 1890.00 (607.00, 4592.75) | 0.29 |
| Uric acid (μmol/L) | 197.10 (129.50, 320.50) | 194.00 (124.00, 314.00) | 206.00 (139.30, 337.25) | 0.083 |
| Glucose (mmol/L) | 8.02 (6.16, 10.80) | 7.99 (6.09, 10.69) | 8.16 (6.36, 10.80) | 0.588 |
| CRP (mg/L) | 44.80 (13.70, 99.80) | 48.00 (13.90, 103.50) | 40.80 (12.25, 93.10) | 0.489 |
| Procalcitonin (ng/mL) | 0.27 (0.10, 1.02) | 0.27 (0.10, 1.02) | 0.26 (0.09, 0.98) | 0.814 |
| IL-6 (pg/mL) | 32.15 (8.09,128) | 33.08 (8.56,130) | 29.13 (7.63,120.98) | 0.944 |
| Platelet (×10 ^9^ /L) | 162.00 (100.50, 239.50) | 162.00 (102.00, 237.00) | 165.50 (95.25, 246.75) | 0.798 |
| Lactate (mmol/L) | 1.30 (1.00, 1.90) | 1.30 (1.00, 1.90) | 1.30 (1.00, 1.80) | 0.706 |
| Calcium (mmol/L) | 2.08 (1.97, 2.19) | 2.08 (1.97, 2.19) | 2.08 (1.98, 2.19) | 0.883 |
| Clinical outcomes |  |  |  |  |
| ICU LOS (days) | 13 (7, 24) | 13 (7, 24) | 12.5 (7.0, 25.0) | 0.732 |
| Hospital LOS (days) | 21.00 (12.00, 33.00) | 21.00 (12.00, 33.00) | 22.50 (12.25, 33.75) | 0.574 |
| ICU mortality (%) | 319 (36.5) | 222 (36.3) | 97 (37.0) | 0.907 |
| Hospital mortality (%) | 361 (41.4) | 251 (41.1) | 110 (42.0) | 0.862 |

Data are shown as median with interquartile range (IQR) for continuous variables or number with percentage for categorical variables.

SCAP: Severe Community-acquired Pneumonia; COPD: chronic obstructive pulmonary disease; n: numbers; IL-6: interleukin-6; BUN: blood urea nitrogen; APTT: activated partial thromboplastin time; PT: prothrombin time; ALT: alanine aminotransferase; AST: aspartate aminotransferase; BNP: Brain Natriuretic Peptide; CRP: C-reactive protein; ICU: intensive care unit; LOS: length of stay.

Table S2. Numbers and percentages of missing values for each variable.

| Variables | Number (percentage) of missing values (total: n=873) |
| --- | --- |
| Demographic characteristics |  |
| Age (years old) | 0 (0%) |
| Sex: male (%) | 0 (0%) |
| Comorbidities |  |
| Cancer history (%) | 0 (0%) |
| Chronic hematological diseases (%) | 0 (0%) |
| Diabetes (%) | 0 (0%) |
| Chronic hepatic diseases (%) | 0 (0%) |
| Chronic renal diseases (%) | 0 (0%) |
| Chronic cardiovascular diseases (%) | 0 (0%) |
| Hypertension (%) | 0 (0%) |
| chronic cerebrovascular diseases (%) | 0 (0%) |
| Vital signs |  |
| Respiratory rate (breath/min) | 51 (6%) |
| Systolic blood pressure (mmHg) | 49 (6%) |
| Diastolic blood pressure (mmHg) | 49 (6%) |
| Temperature (°C) | 49 (6%) |
| Heart rate (beat/min) | 49 (6%) |
| Unconsciousness or insanity | 0 (0%) |
| Laboratory examinations |  |
| White blood cell (×10 ^9^ /L) | 42 (5%) |
| Neutrophil (×10 ^9^ /L) | 42 (5%) |
| Lymphocyte (×10 ^9^ /L) | 42 (5%) |
| Albumin (g/L) | 42 (5%) |
| BUN (mmol/L) | 29 (3%) |
| D-dimer (mg/L) | 60 (7%) |
| Total bilirubin (μmol/L) | 29 (3%) |
| Direct bilirubin (μmol/L) | 29 (3%) |
| Globulin (g/L) | 42 (5%) |
| Monocyte (×10 ^9^ /L) | 47 (5%) |
| Hemoglobin (g/L) | 42 (5%) |
| APTT (s) | 58 (7%) |
| PT (s) | 58 (7%) |
| Fibrinogen (g/L) | 58 (7%) |
| Creatinine (μmol/L) | 42 (5%) |
| Myoglobin (ng/mL) | 128 (15%) |
| ALT (IU/L) | 42 (5%) |
| AST (IU/L) | 42 (5%) |
| Troponin T (ng/L) | 125 (14%) |
| BNP (pg/mL) | 86 (10%) |
| Uric acid (μmol/L) | 42 (5%) |
| Glucose (mmol/L) | 126 (14%) |
| CRP (mg/L) | 144 (16%) |
| Procalcitonin (ng/mL) | 76 (9%) |
| IL-6 (pg/mL) | 166 (19%) |
| Platelet (×10 ^9^ /L) | 42 (5%) |
| Lactate (mmol/L) | 90 (10%) |
| Calcium (mmol/L) | 47 (5%) |

n: numbers; IL-6: interleukin-6; BUN: blood urea nitrogen; APTT: activated partial thromboplastin time; PT: prothrombin time; ALT: alanine aminotransferase; AST: aspartate aminotransferase; BNP: Brain Natriuretic Peptide; CRP: C-reactive protein.
